# Glucocorticoid Receptor β Isoform Predominates in the Human Dysplastic Brain Region and Is Modulated by Age, Sex, and Antiseizure Medication

**DOI:** 10.3390/ijms23094940

**Published:** 2022-04-29

**Authors:** Rosemary Westcott, Natalie Chung, Arnab Ghosh, Lisa Ferguson, William Bingaman, Imad M. Najm, Chaitali Ghosh

**Affiliations:** 1 Cerebrovascular Research, Department of Biomedical Engineering, Lerner Research Institute, Cleveland Clinic, Cleveland, OH 44195, USA; westcor@ccf.org (R.W.); nchung@ramapo.edu (N.C.); 2 Department of Inflammation and Immunity, Lerner Research Institute, Cleveland Clinic, Cleveland, OH 44195, USA; ghosha3@ccf.org; 3 Charles Shor Epilepsy Center, Neurological Institute, Cleveland Clinic, Cleveland, OH 44195, USA; fergusl3@ccf.org (L.F.); bingamb@ccf.org (W.B.); najmi@ccf.org (I.M.N.); 4 Department of Biomedical Engineering and Molecular Medicine, Cleveland Clinic Lerner College of Medicine of Case Western Reserve University, Cleveland, OH 44195, USA

**Keywords:** epilepsy, blood–brain barrier, focal cortical dysplasia, glucocorticoid receptor, cytochrome P450, matrix metalloproteinase, heat-shock protein

## Abstract

The glucocorticoid receptor (GR) at the blood–brain barrier (BBB) is involved in the pathogenesis of drug-resistant epilepsy with focal cortical dysplasia (FCD); however, the roles of GR isoforms GRα and GRβ in the dysplastic brain have not been revealed. We utilized dysplastic/epileptic and non-dysplastic brain tissue from patients who underwent resective epilepsy surgery to identify the GRα and GRβ levels, subcellular localization, and cellular specificity. BBB endothelial cells isolated from the dysplastic brain tissue (EPI-ECs) were used to decipher the key BBB proteins related to drug regulation and BBB integrity compared to control and transfected GRβ-overexpressed BBB endothelial cells. GRβ was upregulated in dysplastic compared to non-dysplastic tissues, and an imbalance of the GRα/GRβ ratio was significant in females vs. males and in patients > 45 years old. In EPI-ECs, the subcellular localization and expression patterns of GRβ, Hsp90, CYP3A4, and CYP2C9 were consistent with GRβ+ brain endothelial cells. Active matrix metalloproteinase levels and activity increased, whereas claudin-5 levels decreased in both EPI-ECs and GRβ+ endothelial cells. In conclusion, the GRβ has a major effect on dysplastic BBB functional proteins and is age and gender-dependent, suggesting a critical role of brain GRβ in dysplasia as a potential biomarker and therapeutic target in epilepsy.

## 1. Introduction

The glucocorticoid receptor (GR) has recently been uncovered as a critical molecular regulator of drug permeability and barrier integrity at the epileptic blood–brain barrier (BBB), where it is found to be overexpressed and to have accelerated maturation [1,2,3]. After alternative splicing of the human GR transcript, multiple isoforms of this receptor are produced, two of the most well-characterized being GRα and GRβ [4,5]. These two GR isoforms differ at the carboxyl terminus [5,6], and due to the splicing at this position, GRβ is not able to bind ligands like glucocorticoids [5,6]. Although GRα is the classic GR isoform—binding glucocorticoids and activating transcription of glucocorticoid receptor element-containing genes—GRβ has shown important implications in inflammation and diseases like rheumatoid arthritis, asthma, and glioma [7,8,9], but the independent roles of GRα and GRβ isoforms have not yet been investigated in epilepsy.

Focal cortical dysplasia (FCD) is a common epilepsy pathology that stems from focal malformations in the cerebral cortex [10], where neuroinflammation is prominent [11,12]. Pharmacoresistance in epilepsy still remains a major clinical challenge, as about one-third of epilepsy patients are non-responsive to antiseizure medications (ASMs) [13,14], and local drug metabolism and efflux activity at the BBB play a critical role in this phenomenon [3,15]. Cytochrome P450 (CYP) drug-metabolizing enzymes and efflux transporters (e.g., P-glycoprotein, Pgp) are functionally important at the BBB and could contribute to pharmacoresistance in epilepsy [1,2,15]. The expression of these enzymes and drug efflux transporters has been found to be regulated by the glucocorticoid receptor (GR) [1,2,3]. The importance of the GRβ isoform has been implicated in other brain disorders, such as glioma, where GRβ plays a critical part in the reactive astrocyte phenotype [9]. However, the specific role of GRβ in the human epileptic brain is not well established and could be an important target for drug regulation and BBB properties in epilepsy.

To identify the involvement of the GRα and GRβ isoforms in FCD, we used cortical brain tissues from patients who underwent surgery for refractory epilepsy to determine: (1) the expression pattern of GRα and GRβ in dysplastic (epileptic) and non-dysplastic (relative control) tissues, (2) changes in GRα/GRβ ratio based on gender and age of these individuals, (3) the expression of neurovascular GR isoform localization in dysplastic vs. non-dysplastic brain tissue, and (4) the subcellular localization of GRα and GRβ based on the ASM combination taken by these patients before surgery as CYP dependent or a partially/independent metabolic pathway. To delineate the involvement of these two GR isoforms in BBB endothelial cells, where GR has been found to have a significant role [1,2,3], we used control primary human brain microvascular endothelial cells, with and without overexpressed GRβ by transfection, and compared that to primary dysplastic human brain endothelial cells (EPI-ECs) isolated from the well-characterized dysplastic brain region. We compared and evaluated the association of GRβ with the subcellular localization and expression levels of other key protein targets involved in drug metabolism and penetration through the BBB (CYP enzymes, P-glycoprotein) and GR regulation (Hsp90). Additionally, in these brain endothelial cells, we investigated the involvement of GRβ in BBB integrity (MMP-9, occludin, claudin-5) and matrix metalloproteinase (MMP) activity, extracellular matrix-degrading proteins responsible for a multitude of events linked to homeostasis and several physiological processes. Together, these data will elucidate the distinct roles of GRα and GRβ in the FCD brain and BBB, providing a deeper understanding of the significance of GR isoforms in epilepsy.

## 2. Results

### 2.1. Decreasing GRα/GRβ Ratio Is Dependent on Age and Gender in Human Dysplastic Brain Tissues

**Figure 1:**
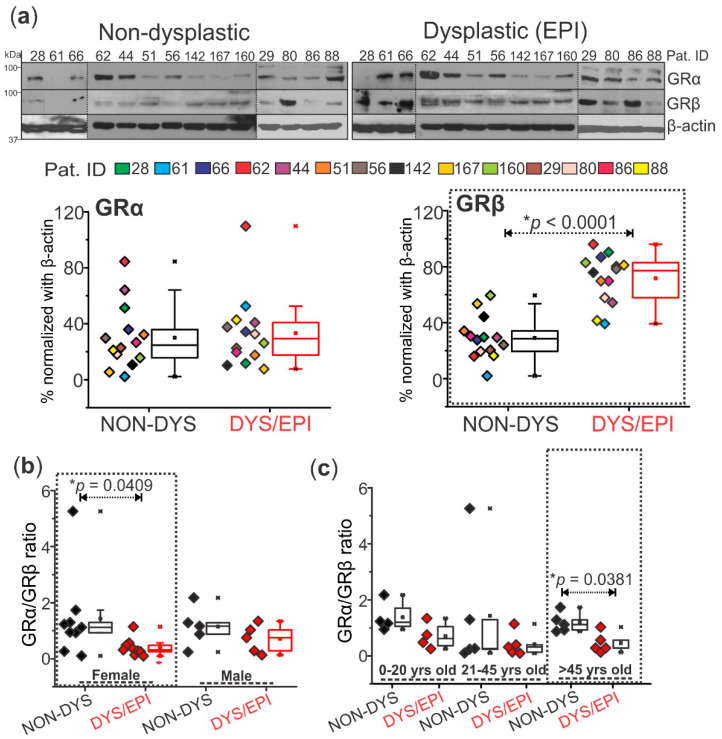
Overexpression of GRβ in the dysplastic brain region compared to non-dysplastic is age and gender-dependent. (**a**) Western blot shows a significant increase (* *p* < 0.0001) in GRβ (~90 kDa) expression in dysplastic (DYS/EPI) compared to non-dysplastic (NON-DYS) brain tissues from patients with FCD (*n* = 14). There was no significant difference in the expression pattern of GRα (~94 kDa) between dysplastic and non-dysplastic tissues. β-actin (~43 kDa) was used as a loading control and for normalization. (**b**) The GRα/GRβ ratio using values obtained from the Western blot in (**a**) was plotted and compared based on the gender of each patient (*n* = 9 females and 5 males). The female group showed a significant decrease (* *p* = 0.0409) in the GRα/GRβ ratio in dysplastic vs. non-dysplastic tissues, corresponding to low GRα and high GRβ levels. There was no significant difference in the GRα/GRβ ratio in dysplastic vs. non-dysplastic tissues in the male patients, implying that GRβ overexpression in the dysplastic region of these patients is gender-dependent. (**c**) Patients from (**a**) were grouped into three age brackets (0–20 years old, 21–45 years old, and >45 years old) based on their age at the time of surgery. The GRα/GRβ ratio followed a decreasing trend with age in dysplastic compared to non-dysplastic brain tissues, and there was a significantly decreased ratio (* *p* = 0.0381) in patients that were above 45 years old. Western blots were performed in duplicate. All values are presented as mean with SD by paired *t*-test.

The cortical brain tissues from dysplastic and non-dysplastic regions from patients with FCD (*n* = 14) revealed significant (* *p* < 0.0001) GRβ overexpression in the dysplastic vs. non-dysplastic brain regions, while GRα expression did not change (Figure 1a). This increase in GRβ expression levels in dysplastic vs. non-dysplastic tissue was found to be dependent on gender. Female patients (*n* = 9) showed a significant decrease in the GRα/GRβ ratio (* *p* = 0.0409) in dysplastic tissues compared to non-dysplastic, a trend that was not observed in male patients (*n* = 5, Figure 1b). Additionally, changes in the GRα/GRβ ratio in dysplastic compared to non-dysplastic brain tissues were also shown to be age-dependent, with a significant decrease in the ratio only being observed in dysplastic tissue of patients over 45 years old compared to non-dysplastic (* *p* = 0.0381, Figure 1c).

### 2.2. Differential Expression and Localization of GRα and GRβ Is Evident in Dysplastic and Non-Dysplastic Human Brain Tissues

**Figure 2:**
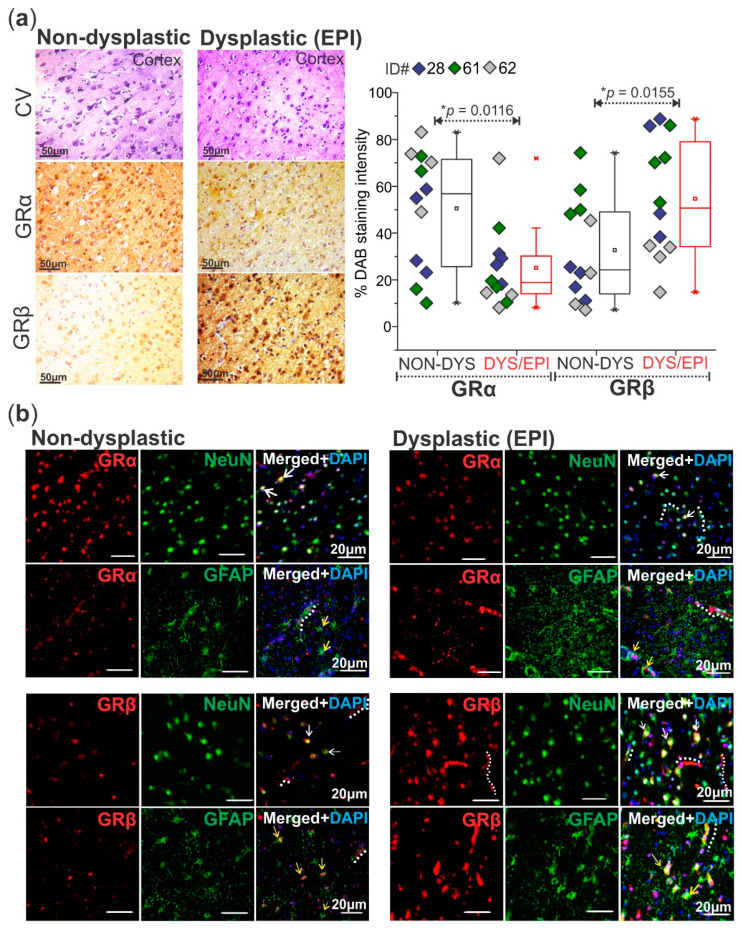
Altered GRα and GRβ expression patterns with differences in neurovascular localization in the dysplastic human brain. (**a**) Cresyl violet (CV) histological staining of the dysplastic (DYS/EPI) vs. non-dysplastic (NON-DYS) cortical regions of a patient with FCD shows dysmorphic neurons and balloon cells in the dysplastic region compared to the relatively normal cortical structure in the non-dysplastic region. Diaminobenzidine (DAB) immunohistochemistry of patient tissues (*n* = 3 patients) confirms increased GRβ isoform levels in the dysplastic brain tissue compared to the non-dysplastic tissue (* *p* = 0.0155). GRα levels are also decreased (* *p* = 0.0116) in the dysplastic tissue compared to non-dysplastic. Images were obtained using a Leica DMIL brightfield microscope. Scale bar = 50 µm. Values are presented as mean with SD by paired *t*-test. (**b**) Immunofluorescent co-staining of GRα and GRβ with neuronal (NeuN) and astrocytic (GFAP) markers in dysplastic (EPI) vs. non-dysplastic human brain tissues (*n* = 3) elucidates the localization of GRα in both the neurons (white arrows) and astrocytes (yellow arrows) in dysplastic and non-dysplastic brain tissue. GRβ immunofluorescent staining shows extensive localization in neurons (NeuN) and astrocytes (GFAP) in the dysplastic tissue in relation to the non-dysplastic tissue. Select microvessels are lined for reference with a dotted white line, where both GRα and GRβ immunostaining is evident. Images were obtained using a Leica DMIL LED microscope with a gain of 1.0. Scale bar = 20 μm.

The histology of cortical brain tissues resected from the epileptic lesion (dysplastic) and from the surrounding, relatively normal brain area (non-dysplastic) was confirmed by cresyl violet staining and visualization of dysmorphic neurons, characteristic of FCD pathology (*n* = 3 patients, Figure 2a). In these same patients, DAB staining of both GRα and GRβ isoforms showed that, in general, GRβ levels were significantly elevated (* *p* = 0.0155) and GRα levels significantly decreased (* *p* = 0.0116) in the dysplastic brain region compared to the non-dysplastic region in these patients (Figure 2a). Co-immunohistochemistry of GRα with NeuN (neuronal nuclei marker) and GFAP (glial fibrillary acidic protein) confirmed the presence of GRα in the neurons and astrocytes of both the dysplastic and non-dysplastic brain regions (Figure 2b). GRβ staining was most prominent in the neurons and of the dysplastic tissues and scattered in the astrocytes (Figure 2b). Both GR isoforms were consistently expressed in the microvessels of dysplastic brain tissues in the cortex, marked with dotted lines in a few locations for reference (Figure 2b).

### 2.3. Antiseizure Drug Combination Regulates Subcellular Localization of GRα and GRβ Isoforms in Human Cortical Brain Tissue

**Figure 3:**
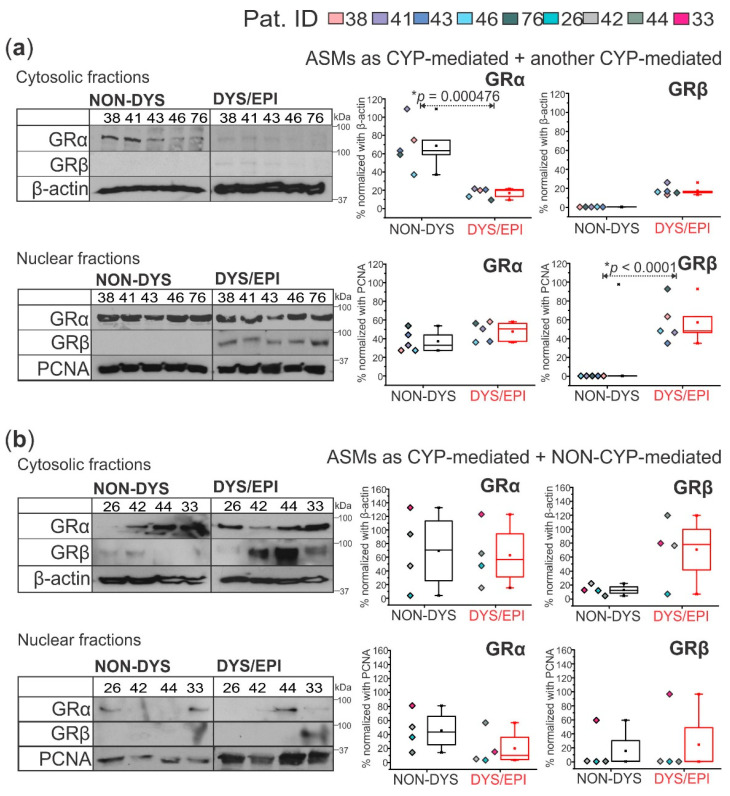
GRα and GRβ subcellular localization in human dysplastic brain tissues is dependent on the antiseizure medication (ASM) combination. (**a**) In patients who took a combination of two or more cytochrome P450 (CYP)-mediated ASMs (*n* = 5 patients), cytoplasmic GRα (~94 kDa) was significantly decreased (* *p* = 0.000476) and nuclear GRβ (~90 kDa) significantly elevated (* *p* < 0.0001) in dysplastic compared to non-dysplastic tissues. (**b**) In patients who took a combination of CYP-mediated and NON-CYP-mediated ASMs (*n* = 4 patients), there were no significant differences between GRα or GRβ expression in dysplastic and non-dysplastic tissues in either the cytoplasm or nucleus. β-actin (~43 kDa) and PCNA (~35 kDa) were used as loading controls for the cytosolic and nuclear fractions, respectively, and for normalization. Western blots were performed in duplicate. All values are presented as mean with SD by one-way ANOVA with a Tukey post hoc test.

The cytosolic fraction of cortical brain tissue from the non-dysplastic regions of FCD patients taking ASMs as two or more CYP-mediated medications (*n* = 5) shows a significant increase (* *p* = 0.000476) in cytosolic GRα expression compared to the dysplastic tissue (Figure 3a). Compared to the nuclear fraction, there are significantly greater GRα levels (* *p* = 0.0256) in the cytosolic fraction in the non-dysplastic tissue of the CYP-mediated + CYP-mediated ASMs group, which is opposite to what is observed in the dysplastic tissue, showing significantly increased nuclear GRα (* *p* = 0.0306). Additionally, the nuclear fractions from these same tissues revealed increased levels of GRβ in dysplastic tissues compared to non-dysplastic (* *p* < 0.0001, Figure 3a). In the nuclear fraction of dysplastic tissues from this group, GRβ is significantly increased (* *p* = 0.000266) compared to the dysplastic cytosolic fraction, but between the non-dysplastic cytosolic and nuclear fractions, there is no difference in GRβ expression. The cytosolic and nuclear fractions of brain tissues from patients taking ASM combinations as CYP+NON-CYP-mediated (*n* = 4) showed a different pattern of GRα and GRβ localization. There is no significant difference in GRα or GRβ expression between non-dysplastic and dysplastic tissues of this group in either subcellular fraction or between the cytosolic and nuclear fractions with both GR isoforms evaluated (Figure 3b).

### 2.4. GRβ Overexpression in Human Brain Microvascular Endothelial Cells Regulates Expression and Subcellular Localization of Critical BBB Proteins and MMP Activity

**Figure 4:**
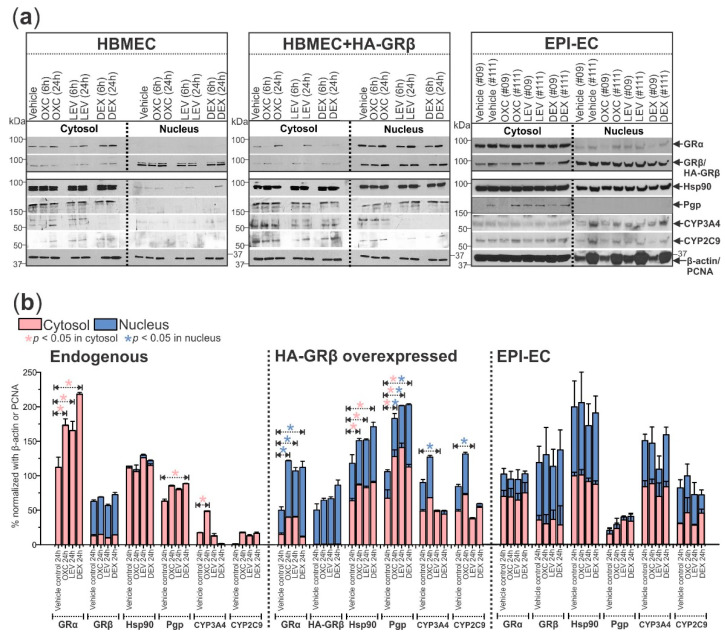
GRβ alters the expression and subcellular localization of GRα and other key drug regulatory-related proteins in human brain endothelial cells. (**a**) Protein targets critical for drug metabolism and transport at the BBB showed altered expression and subcellular localization patterns in HBMEC+HA-GRβ (*n* = 3) and EPI-ECs (*n* = 2) compared to HBMECs (*n* = 3) with endogenous GRβ levels by Western blot. Quantification is shown in (**b**). GRα (~94 kDa) was only present in the cytosol of HBMECs but was highly present in the nuclear fraction of HBMEC+HA-GRβ. EPI-ECs showed a pattern of GRα subcellular localization that was a mixture of what was seen in HBMECs and HBMEC+HA-GRβ, with expression in the cytosol and nucleus. Oxcarbazepine (OXC), levetiracetam (LEV), and dexamethasone (DEX) treatments all significantly increased the expression of GRα after 24 h in the cytosol of HBMECs and in the nuclear fraction of HBMEC+HA-GRβ but caused no change in GRα expression in EPI-ECs. After HA-GRβ overexpression in HBMECs, GRβ (~90 kDa) was exclusively localized in the nucleus and negligible in the cytosol, but in EPI-ECs it was present in both the cytoplasmic and nucleus. Also, Hsp90 (~90 kDa) was almost explicitly seen in the cytosolic fraction of HBMECs, but HA-GRβ overexpression caused Hsp90 to be found in the nuclear fraction as well as the cytosolic, which is consistent with what was observed in EPI-ECs. Pgp (~170 kDa) was only expressed in the cytosol of HBMECs and EPI-ECs but in the nucleus of HBMEC+HA-GRβ. After OXC, LEV, or DEX treatment for 24 h, Pgp levels in the cytosol and nucleus were increased in HBMEC+HA-GRβ; although, only 24 h DEX treatment increased cytosolic Pgp expression in HBMECs. CYP3A4 (~57 kDa) and CYP2C9 (~59 kDa) levels in the cytosol and nucleus were both significantly lower in HBMECs with endogenous GRβ compared to HBMEC+HA-GRβ (CYP3A4 cytosolic: * *p* < 0.0001, CYP3A4 nuclear: * *p* = 0.000487, CYP2C9 cytosolic: * *p* < 0.0001, CYP2C9 nuclear: * *p* = 0.000161) and EPI-ECs (CYP3A4 cytosolic: * *p* < 0.0001, CYP3A4 nuclear: * *p* < 0.0001, CYP2C9 cytosolic: * *p* < 0.0001, CYP2C9 nuclear: * *p* < 0.0001). Although 24 h OXC treatment significantly increased cytosolic CYP3A4 levels in HBMECs, 24 h LEV treatment significantly decreased nuclear CYP3A4 and CYP2C9 levels in HBMEC+HA-GRβ. EPI-ECs show elevated levels of both of these CYP enzymes compared to HBMECs, but drug treatment did not affect expression levels. β-actin (~43 kDa) and PCNA (~35 kDa) were used as loading controls for the cytosolic and nuclear fractions, respectively, and for normalization. Western blots were performed in duplicate. All values are presented as mean with SD by two-way ANOVA with a Tukey post hoc test.

The human brain microvascular endothelial cells (HBMECs, *n* = 3) transfected with HA-tagged GRβ (HBMEC+HA-GRβ, *n* = 3) and dysplastic/epileptic endothelial cells (EPI-ECs, *n* = 2) evaluated by Western blot (shown by the representative blots, Figure 4a, and quantification in Figure 4b) show changes in the subcellular localization of the GR isoforms and a heat-shock protein chaperone (Hsp90) critical for GR maturation and function, compared to HBMECs (non-transfected control group, Figure 4). In HBMEC controls, GRα was only found in the cytosol, whereas in cells with overexpressed GRβ, GRα was found mostly in the nuclear fraction (Figure 4). In EPI-ECs, GRα was found in the cytoplasmic and partially in the nuclear fractions and was not significantly impacted by drug treatment. After GRβ overexpression in HBMECs, GRα levels increased in the nucleus after OXC, LEV, or DEX treatment for 24 h but not in the cytosol (Figure 4), which is reversed in the case of HBMEC/control endothelial cells (non-transfected). In all three cell types, the GRβ localization remained most prominent in the nuclear fraction and was unaffected by drug treatment (Figure 4). In both the cytosolic and nuclear fractions, EPI-ECs showed the highest GRβ expression of the three cell types, determined by the two-way ANOVA group effect. Hsp90 expression was increased in the cytosol after 24 h of OXC, LEV, or DEX treatment but only in HBMEC+HA-GRβ (Figure 4). Hsp90 was not extensively found in the nucleus of HBMECs. However, besides the cytosolic fractions, Hsp90 expression was also prominent in the nuclear fraction in HBMEC+HA-GRβ (* *p* = 0.000152) and EPI-ECs (* *p* < 0.0001) compared to HBMECs, analyzed by two-way ANOVA group effect.

The expression changes in the three cell types and with drug treatment were also evaluated for downstream proteins relating to drug efflux activity and local drug metabolism at the BBB. Pgp was found in the cytosolic fraction in HBMEC and EPI-EC but was only observed in the nuclear fraction in HBMECs with GRβ overexpression. Pgp expression in the HBMEC and HBMEC+HA-GRβ increased after 24 h of each drug treatment—OXC, LEV, and DEX (Figure 4). Cytosolic and nuclear CYP3A4 and CYP2C9 expression was significantly increased in HBMEC+HA-GRβ (CYP3A4 cytosolic: * *p* < 0.0001, CYP3A4 nuclear: * *p* = 0.000487, CYP2C9 cytosolic: * *p* < 0.0001, CYP2C9 nuclear: * *p* = 0.000161) and EPI-ECs (CYP3A4 cytosolic: * *p* < 0.0001, CYP3A4 nuclear: * *p* < 0.0001, CYP2C9 cytosolic: * *p* < 0.0001, CYP2C9 nuclear: * *p* < 0.0001) compared to HBMECs, according to two-way ANOVA group effect. A 24 h LEV treatment significantly decreased the nuclear expression of both CYP enzymes in HBMEC+HA-GRβ (CYP3A4: * *p* = 0.0415, CYP2C9: * *p* = 0.0355), while 24 h OXC treatment significantly increased CYP3A4 levels (* *p* = 0.0268) in the cytosol in HBMEC, both compared to their respective vehicle controls at 24 h (Figure 4a,b).

**Figure 5:**
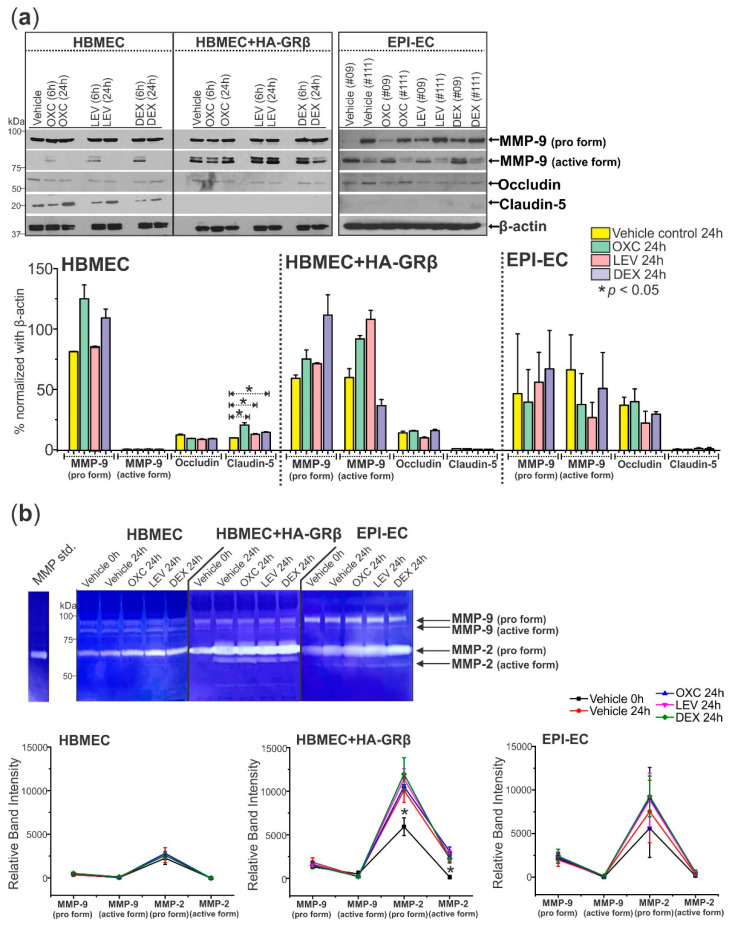
Overexpressed GRβ in human brain endothelial cells and EPI-ECs increases active MMP-9 protein levels, decreases Claudin-5 expression, and increases MMP activity. (**a**) HBMEC+HA-GRβ shows increased levels of the active form of MMP-9 (~82 kDa) compared to HBMECs with endogenous GRβ but no change in the levels of the pro form (~92 kDa) of this protein. Occludin (~65 kDa) levels remain unchanged with drug treatment and GRβ expression levels, but 24 h treatment of OXC (* *p* < 0.0001), LEV (* *p* = 0.0361), or DEX (* *p* = 0.000918) increased Claudin-5 (~18 kDa) levels in HBMECs but not HBMEC+HA-GRβ or EPI-ECs. β-actin (~43 kDa) was used as a loading control and for normalization. (**b**) Representative MMP activity shown by gelatin zymography. Quantification of HBMEC+HA-GRβ overexpressed cells showed significantly elevated MMP-2 pro and active forms (* *p* < 0.0001 for both) compared to HBMECs with endogenous GRβ. Similar changes in MMP activity were seen in EPI-ECs compared to HBMECs (pro MMP-9: * *p* = 0.00193, pro MMP-2: * *p* < 0.0001, active MMP-2: * *p* = 0.0180). Western blots were performed in duplicate. All values (**a**,**b**) are presented as mean with SD by two-way ANOVA with a Tukey post hoc test.

GRβ overexpression and drug treatment also altered the levels of protein targets responsible for BBB integrity. There was significantly more active MMP-9 expression in HBMEC+HA-GRβ (* *p* < 0.0001) and EPI-ECs (* *p* = 0.000174) compared to HBMECs (Figure 5a). However, drug treatment minimally impacted the pro MMP-9 (*p* = 0.0880) and active MMP-9 (*p* = 0.300) levels, evaluated by the two-way ANOVA treatment group effect. Occludin levels were also non-significantly changed within GRβ overexpression compared to non-transfected HBMECs (*p* = 0.252) and were also not affected by drug treatment. In contrast, claudin-5 levels were significantly decreased in EPI-ECs (* *p* < 0.0001) and HBMEC+HA-GRβ (* *p* < 0.0001) compared to HBMECs. In HBMECs, claudin-5 levels were significantly increased after 24 h of OXC (* *p* < 0.0001), LEV (* *p* = 0.0361), and DEX (* *p* = 0.000918) treatment compared to the vehicle control (Figure 5a). Drug treatment did not affect claudin-5 levels in HBMEC+HA-GRβ and EPI-ECs (Figure 5a).

In addition to changing expression levels, the activity of MMPs (MMP-2 and MMP-9) also showed alterations between cell types but were further unaffected by drug treatment (Figure 5b). Pro MMP-9, pro MMP-2, and active MMP-2 activity were all significantly increased in HBMEC+HA-GRβ (pro MMP-9: * *p* < 0.0001, pro MMP-2: * *p* < 0.0001, active MMP-2: * *p* < 0.0001) and EPI-ECs (pro MMP-9: * *p* < 0.0001, pro MMP-2: * *p* < 0.0001, active MMP-2: * *p* = 0.0180) compared to HBMECs.

## 3. Discussion

GR has already proven to be an important player in drug-resistant epilepsy due to focal cortical dysplasia (FCD), but the individual roles and clinical significance of the brain GRα and GRβ isoforms in FCD have not been well defined. The current study identifies for the first time that upregulated GRβ or a decreased GRα/GRβ ratio in the dysplastic brain could contribute to pathogenesis and drug response in pharmacoresistant epilepsy, particularly in certain subsets of patients, such as females and those over 45 years old. By recognizing the imbalance of these two GR isoforms in the dysplastic brain region compared to an adjacent relatively non-dysplastic region, which could be a biomarker of the dysplastic focus, and the effect of GRβ on drug response in BBB endothelial cells, the mechanism underlying the BBB involvement in drug-resistant epilepsy is further unveiled.

We found that of the two GR isoforms, GRβ is overexpressed in the dysplastic brain region compared to a non-dysplastic brain region of the same patients (Figure 1), whereas GRα isoform expression was not significantly overexpressed in the dysplastic brain. Previously, our group discovered that total GR is overexpressed in the dysplastic brain [1,2,3], suggesting that a great proportion of GR overexpression is possibly due to GRβ based on these novel findings. Although the expression and ratio of GRα and GRβ isoforms in epilepsy have not yet been reported until now, interestingly, this information has been found to be pertinent to other diseases such as asthma, rheumatoid arthritis, and glioma [7,8,9]. Sex and age differences relating to GR have been implicated in inflammatory bowel disease (IBD) as well [16] which is consistent with our case, where a significant difference in the dysplastic vs. non-dysplastic GRα/GRβ ratio in female patients and in patients > 45 years old was identified. Reports also indicate that female patients with IBD were more likely to develop a dependence on glucocorticoid treatment compared to male patients, who were less likely to relapse after glucocorticoid dose reduction [16]. In general, sex differences have been identified in epilepsy, with FCD being more common in male pediatric epilepsy patients compared to females [17]. The relationship between a decreased GRα/GRβ ratio in female patients compared to males in this study would be interesting to further investigate, such as the possibility that females with a lower GRα/GRβ ratio could be more susceptible to the development of focal cortical dysplasia in epilepsy. This could be further investigated. Additionally, age played a statistically significant role in glucocorticoid response in these IBD patients, with glucocorticoid-resistant patients being older than responders [16]. GRβ is known to be involved in glucocorticoid resistance in multiple diseases [18,19], so the current findings of GRβ overexpression and a dependency of sex and age in the GRα/GRβ ratio in dysplastic compared to non-dysplastic brain tissue are consistent with findings in other disorders related to inflammatory factors. Treatment with corticosteroids, such as prednisolone, has been successful in managing seizures in some pediatric epilepsy patients [20,21], although steroid treatment for older adult epilepsy has not been as extensively studied. Steroid treatment in older patients with focal cortical dysplasia may not be as successful due to the decreased ratio of GRα/GRβ, possibly contributing to glucocorticoid resistance.

In glioma, GRβ was also found to be overexpressed in the nuclei of injured astrocytes, where it was associated with β-catenin. After GRβ downregulation, the reactive astrocyte phenotype seen in glioma was dampened, showing that overexpression of this particular GR isoform is functionally relevant to the disease phenotype and pathogenesis of glioma [9]. In terms of epilepsy, GRβ overexpression could have similar effects at multiple levels of the neurovasculature. We were able to detect GRβ overexpression predominantly in the dysplastic microvessels, astrocytes, and neurons, and GRα was also located in these cell types, which was expected due to the importance of glucocorticoid signaling in brain function and regulation in these cells [22,23,24]; although this isoform was not as robustly expressed as GRβ which is clearly distinguishable within dysplastic and non-dysplastic brain regions. It has been previously implicated that a decreasing GRα/GRβ ratio (lower GRα and higher GRβ levels), which is what we see in this current study, relates to the ability of GRβ to act as a dominant negative regulator of GRα function [25,26]. 

In addition to expression changes, the drug regimen of the FCD patients affected the subcellular localization of GRα and GRβ isoforms levels (Figure 3). Previous studies have shown that GR is the upstream regulator of CYP3A4, CYP2C9, and Pgp expression [1,2]. Here, the GR isoform subcellular localization was followed in individuals that received multiple CYP-mediated ASMs vs. a combination of CYP+NON-CYP-mediated ASMs within the dysplastic and non-dysplastic brain regions. In the dysplastic tissue of the CYP+CYP group, both GRα and GRβ were primarily located in the nuclear fraction, the functionally active location. Both GR isoforms in the CYP+NON-CYP ASM group trended more towards the cytosolic fraction in the dysplastic and non-dysplastic tissues. GRα moves to the nucleus only after ligand binding, so the CYP+CYP ASM combination could trigger faster GR maturation and nuclear translocation through a drug-dependent mechanism that is not present with CYP+NON-CYP ASM combinations, possibly facilitated by heat-shock protein interaction with GR [3,27,28].

With that finding in mind, we further asked whether GRβ was the governing GR isoform that caused the changes in subcellular localization with drug treatment in EPI-ECs. Our overall goal was to determine if the expression and subcellular localization changes in targets important for BBB drug regulation observed between EPI-ECs and control endothelial cells could be attributed to GRβ overexpression. One possible explanation for increased nuclear GRα levels in endothelial cells with overexpressed GRβ could be that GRβ either drives GRα into the nucleus or traps it there, possibly modulating the downstream events differently in a disease state; however, this warrants further investigation. Because GRβ expression itself is driven by other factors, like cytokine levels, and it does not bind ligands [5,6], it is possible that drug monotherapy does not play as much of a role in its expression and nuclear translocation as polytherapy, as seen in Figure 3. In terms of GR regulation, Hsp90 is a major target for GR maturation and nuclear translocation [27,28]. However, Hsp90 does not only interact with the GRα isoform. It has been previously shown that Hsp90 is essential for GRβ nuclear translocation and that increased nuclear Hsp90 levels correspond with GRβ overexpression in glaucomatous trabecular meshwork cells [29]. In EPI-ECs and HBMEC+HA-GRβ, there were increased nuclear levels of Hsp90 compared to HBMEC. In other reports, nuclear Hsp90 accumulation was reported to be positively associated with metastasis and negatively associated with survival in patients with non-small cell lung cancer [30]. Interestingly, we show for the first time that GRβ overexpression contributes to the nuclear accumulation of Hsp90 in epilepsy. Hsp90 accumulation in the nucleus relating to GRβ overexpression could have clinical relevance in epilepsy which should be further investigated.

The subcellular localization of Hsp90 could be related to GR maturation, which is associated with the expression of downstream targets, such as cytochrome P450 enzymes CYP3A4 and CYP2C9. Both of these CYP isoforms had elevated expression in EPI-ECs and in HBMEC+HA-GRβ, with localization in both the cytosol and nucleus, which suggests that these CYP enzymes are functional in the nucleus in epileptic endothelial cells, which is associated with GRβ overexpression. CYP1B1, another CYP isoform, mRNA has also been previously found in the cytoplasm and nucleus of human neurons and astrocytes in the cortex of the brain; although, the nuclear function remains unclear [31]. The role of nuclear CYP3A4 and CYP2C9 in epilepsy relating to GRβ overexpression needs to be explored in the future.

In both EPI-ECs and HBMEC+HA-GRβ, unlike claudin-5, occludin levels were not significantly changed by drug treatment and remained relatively similar among the three cell types. Claudin-5 expression increased after 24 h of treatment with OXC, LEV, and DEX in HBMEC control cells [32,33] but not in HBMEC+HA-GRβ or EPI-ECs. This discrepancy could allude to the notion that, in this scenario, claudin-5 levels could not be rescued by pharmacological treatment due to the high levels of GRβ in the non-responding cells. In a study done with bone marrow-derived macrophages, LPS-induced resistance to DEX treatment was attributed to a 7-fold increase in GRβ mRNA levels in these cells [34], which could explain the resistance to DEX-mediated claudin-5 increase in HBMEC+HA-GRβ or EPI-ECs in our study. Interestingly, a recent study has also found that claudin-5 expression was significantly decreased 1.97-fold in epileptic brain microvessels compared to controls, but occludin expression was not significantly different between the two groups [35]. This supporting evidence further confirms that the decrease in tight junction proteins in the epileptic brain region may not afflict all types of tight junction proteins, and we describe here for the first time that GRβ may play a role in that phenomenon.

Tight junction proteins can also be broken down by matrix metalloproteinases (MMPs), like MMP-9 [36], exacerbating the epileptic condition. Not only can MMP-9, as a calcium-dependent zinc-containing endopeptidase critical for neurovascular homeostasis, afflict BBB damage, but it also affects neuronal function [37,38]. Increased expression of the active form of MMP-9 and increased MMP-2 activity in HBMEC+HA-GRβ and EPI-ECs was not significantly affected by drug treatment (OXC, LEV, or DEX for 24 h), which could imply that MMP function in the dysplastic BBB is a pathological issue that is not rescued or worsened by drug treatment. GRβ overexpression has been found to enhance the expression of tumor necrosis factor-α (TNF-α) in a human monocyte cell line [39], and similarly, MMP-9 expression has been found to be increased by TNF-α in a human epithelial cell line [40]. The increase in MMP expression and activity observed in HBMEC+HA-GRβ and EPI-ECs could be related to cytokine production, such as TNF-α, which is likely mediated by GRβ overexpression.

A role for GR isoforms has been implicated in components of the blood, such as monocytes and platelets, in major depressive disorder [39] and immune thrombocytopenia [41,42]; although, there is little to no evidence of GR isoforms in the blood of epilepsy patients. Clinical studies have shown neuronal migration disorders in the lesioned vs. non-lesional patients and detected epileptogenicity and have shown to produce clustered magnetoencephalography spike sources under total intravenous anesthesia. It would be interesting to investigate whether the GRα/GRβ difference that was observed in the dysplastic brain tissue compared to the non-dysplastic brain tissue of these patients could be detected in the blood of epilepsy patients as a disease biomarker. 

**Figure 6:**
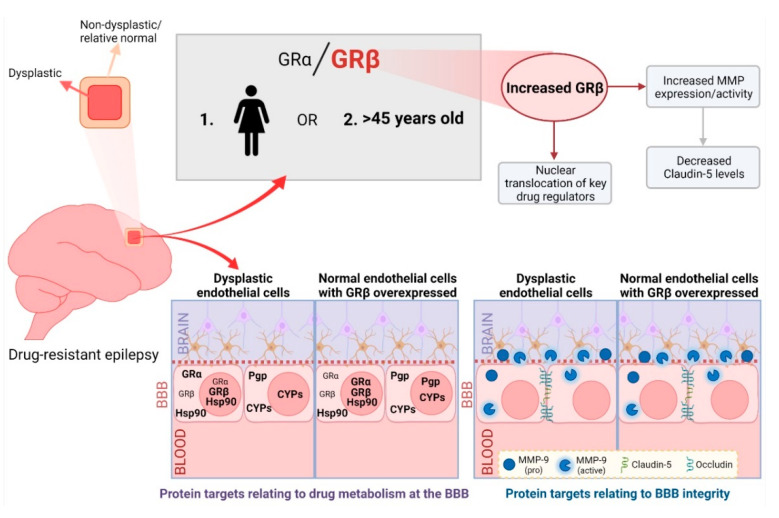
Summarizing the importance of GRβ overexpression in the dysplastic brain. We found an imbalance of GRα and GRβ, with increased GRβ levels, in the dysplastic brain region compared to a non-dysplastic region in patients with focal cortical dysplasia, particularly in females or individuals greater than 45 years old. The GR isoform imbalance, with GRβ being dominant in the dysplastic brain region, causes changes in the subcellular localization and expression patterns of critical BBB proteins related to drug regulation and BBB integrity as well as MMP activity in dysplastic endothelial cells. This is confirmed by overexpressing GRβ in normal brain microvascular endothelial cells which is found more comparable to dysplastic conditions. Delineating the role of GRβ in the dysplastic brain brings us one step closer to improved targeted therapy for epilepsy patients with focal cortical dysplasia. Figure created with BioRender.com (accessed on 6 April 2022).

In conclusion, the GRα/GRβ imbalance that is observed in the dysplastic tissue of patients, particularly females and those above 45 years old, compared to the non-dysplastic tissue could be a critical marker of the diseased brain region. While GRβ overexpression in brain microvascular endothelial cells altered the subcellular localization and expression of multiple protein targets vital to the proper functioning of the neurovasculature (summarized in Figure 6). Delineating the predominant GR isoform in the dysplastic region could allow for future isoform-specific targeting that would be critical for BBB functional homeostasis in the dysplastic brain region and better-targeted therapy for patients with drug-resistant epilepsy.

## 4. Materials and Methods

### 4.1. Ethical Approval

Informed consent was obtained from patients prior to tissue procurement under a Cleveland Clinic Institutional Review Board-approved protocol (IRB #07-322). This study was compliant with the principles outlined in the Declaration of Helsinki, and the authors understand the ethical principles. Brain specimens from both male and female subjects (*n* = 23) with pharmacoresistant epilepsy were obtained following focal surgical resections. Brain tissues from epileptic/dysplastic (DYS/EPI) and non-dysplastic/relative normal (NON-DYS) regions were resected after prior non-invasive (scalp video-EEG monitoring, magnetic resonance imaging, and positron emission tomography) and invasive (stereo-electro encephalography) evaluations. The non-dysplastic resected tissue region from each subject was considered as an internal relative control to the dysplastic tissue. The experimental outline is provided in Figure S1. Additional patient information (age, gender, ASMs, seizure frequency, epilepsy duration, resected tissue region, pathology, and experimental use of tissue) is summarized in Table 1.

### 4.2. Tissue Lysate Preparation and Fractionation

Approximately 50 mg of fresh-frozen human cortical tissue resected from patients with drug-resistant epilepsy due to FCD (*n* = 14) was lysed with radioimmunoprecipitation assay (RIPA; Sigma-Aldrich, Burlington, MA, USA, cat. R0278) buffer combined with 1× protease inhibitor cocktail (Sigma, cat. P8340) as previously described [3,15]. To obtain cytoplasmic and nuclear fractions of the tissue, 50 mg of fresh-frozen tissue (*n* = 9 patients) was fractionated using the NE-PER Nuclear and Cytoplasmic Extraction Reagents kit (Thermo Fisher Scientific, Waltham, MA, USA, cat. 78833) according to the manufacturer’s instructions and as previously described [1]. The protein concentration of the lysates was estimated by the Bradford method.

### 4.3. Western Blotting

For the human brain tissue lysates, GRα and GRβ were separated by 8% sodium dodecyl sulfate polyacrylamide gel electrophoresis (SDS-PAGE) and later transferred to polyvinylidene fluoride (PVDF) membranes (EMD Millipore Corp., Burlington, MA, USA, cat. IPVH00010) by semidry transfer (Trans-Blot© SD Semi-Dry Transfer Cell, Bio-Rad, Hercules, CA, USA). In brief, the membranes were probed overnight at 4 °C with the respective primary antibody followed by the appropriate secondary antibody for 1 h at room temperature (Supplementary Table S1), as previously described [15]. For the target proteins, the PVDF membranes were incubated in stripping buffer (Thermo Scientific, cat. 21059) for 20 min at room temperature followed by blocking of the membranes in 5% milk for 4 h before re-probing. In each case, the protein expression was normalized by β-actin (total lysate and cytoplasmic fractions) or proliferating cell nuclear antigen (PCNA, nuclear fractions) as loading controls, and the densitometric quantification of the images was performed using ImageJ software (National Institute of Health, Bethesda, MD, USA). Western blot using cell lysates and subcellular fractions for GRα, GRβ, Hsp90, Pgp, CYP3A4, CYP2C9 (8% gels) MMP-9, occludin, and claudin-5 (10% gels) were performed in a similar manner as stated above. Antibody information can be found in Table S1 and full representative blots in Figure S2.

### 4.4. Histology by Cresyl Violet Staining

Gross anatomical evaluation of brain tissue specimens from patients who had undergone surgical resection for intractable epilepsy due to FCD from dysplastic compared to the respective non-dysplastic region was performed by cresyl violet histological staining on brain slices (*n* = 3 patients, 5 sections each) for observation of the cellular structures to identify dyslamination, ectopic neurons, and vascular malformations [43].

### 4.5. Diaminobenzidine Staining

Brain sections (*n* = 3 patients, 5 sections each) were permeabilized (0.3% TWEEN in 0.1 M PBS), blocked for endogenous peroxidase (0.3% hydrogen peroxide in methanol) and non-specific staining (5% normal goat serum in 0.1 M PBS + 0.4% Triton-X 100), and incubated at 4 °C overnight with GRα or GRβ primary antibodies. The detailed method has been described previously [43,44]. After washing, the sections were incubated for 1 h at room temperature with the respective biotinylated secondary antibody followed by 1 h with the avidin/biotin complex (Vector Labs, Burlingame, CA, USA, Elite Vectastain ABC kit, cat. PK-6102), visualization with diaminobenzidine (DAB) (Vector Labs, peroxidase substrate kit, SK-4100; nickel omitted), dehydration, and mounting with Permount (Thermo Fisher Scientific, cat. SP15-500). Primary and secondary antibodies used are listed in Table S1. Images were obtained by bright field microscopy using a Leica DMIL microscope and Q Capture for image acquisition. Quantification of the DAB staining (*n* = 4 images/patient) was performed using ImageJ software (National Institutes of Health). The background was removed using the brightness and contrast controls and the Rolling Ball Radius function. Images were converted to 8-bit, and the threshold was maintained by using the Adjust Threshold function. The resulting highlights after adjustment were then measured for average relative DAB intensity using the Measure function. Origin Pro 9.0 Software (version: 90E, Origin Lab Corp., Northampton, MA, USA) was then utilized to identify significant differences in expression between the dysplastic and non-dysplastic brain tissue regions.

### 4.6. Immunofluorescence Staining

We also determined the expression and localization patterns of these two GR isoforms by immunofluorescence staining on contiguous brain slices (*n* = 4 patients, 5 sections each). The slices were immunostained for GRα and GRβ. Astrocytic (GFAP: glial fibrillary acidic protein) and neuronal (NeuN: neuronal nuclei) markers were also used to identify the cellular localization of the two GR isoforms. The concentrations and sources of all primary and secondary antibodies used are listed in Supplementary Table S1. After blocking for 1 h, the sections were incubated with the targeted primary antibody overnight at 4 °C followed by the respective secondary antibody for 2 h at room temperature. The tissues were blocked for autofluorescence with Sudan Black prior to mounting with VECTASHIELD^®^ Mounting Medium with DAPI (Vector Laboratories, cat. H-1200). Images were acquired by fluorescence microscopy using a Leica DMIL LED microscope with a gain of 1.0. The acquired images were processed using ImageJ software. Antibody information can be found in Table S1.

### 4.7. Primary Brain Endothelial Cell Culture

We used primary microvascular endothelial cells derived from brain specimens resected from patients with drug-resistant epilepsy (human epileptic endothelial cells (EPI-ECs), *n* = 2), as described earlier [44], obtained from EPI brain regions. Briefly, resected brain tissue specimens were incubated in collagenase type II (2 mg/mL; Thermo Fisher Scientific, cat. 17101-015) at 37 °C for 40 min to dissociate the ECs. The collagenase was then washed with endothelial cell medium 1.5 g/100 mL MCDB-105 (Sigma-Aldrich, cat. M6395), 15 mg/100 mL endothelial cell growth supplement (EMD Millipore, cat. 02-102), 800 U/100 mL heparin (Sigma-Aldrich, cat. H3149), 10% fetal bovine serum (Atlas Biologicals, Fort Collins, CO, USA, cat. F-0500-DR), and 1% penicillin/streptomycin), and the dissociated cells were plated initially in fibronectin-coated (Sigma-Aldrich, cat. F4759; 3 μg/cm^2^) 75 cm^2^ tissue culture flasks [1,2]. Primary control human brain microvascular endothelial cells (HBMECs) were purchased from Cell Systems (Kirkland, WA, USA, cat. ACBRI 376). The HBMECs were used as a control compared to the EPI-ECs and for transfection. According to the company, the HBMECs were dissociated from normal human brain cortical tissue obtained from healthy donors using a Beckman elutriation system and characterized by von Willebrand factor staining. Other specific details are available on the company website (Cell Systems, https://cell-systems.com/products/human-brain-microvascular-endothelial-cells-acbri-376?variant=37945739019 [accessed on 28 January 2022]). All cell culture treatment experiments were performed in 100 mm Petri dishes at 70–80% confluency.

### 4.8. Overexpression of HA-GRβ by Transfection

To simulate the increase in GRβ expression observed in the dysplastic tissue compared to non-dysplastic, HBMECs were transfected with HA-tagged GRβ DNA (1.075 µg/µL). The custom HA-tagged GRβ plasmid was obtained from OriGene Technologies utilizing the open reading frame (ORF) from cat. RC220377 (OriGene Technologies, Rockville, MD, USA) cloned in a pCMV6-AC-HA vector (OriGene Technologies, cat. PS100004). To achieve this transfection, 5 µg of HA-GRβ DNA was mixed in serum-free Dulbecco’s modified eagle medium (DMEM/F12) and was later combined with a 30 µg mixture of lipofectamine (Thermo Fisher Scientific, cat. 18324-012) in serum-free DMEM to form the DNA+lipofectamine complex. This mixture was set aside for 25 min at room temperature. Once formed, this DNA+lipofectamine complex was combined with additional serum-free DMEM and added to the 100 mm Petri dish of 70% confluent HBMECs and left to incubate for 5 h at 37 °C. After the incubation was complete, the serum-free media containing the DNA+lipofectamine complex was aspirated, the plate was washed with 0.1 M phosphate-buffered saline (PBS), and the PBS was replaced with normal HBMEC media (Cell Systems, cat. 4Z0-500) until the following day when subsequent drug treatment experiments were performed. These transfected cells will be denoted throughout as “HBMEC+HA-GRβ”.

### 4.9. Drug Treatment with Cellular Fractionation

To determine the effect of ASM (oxcarbazepine: OXC or levetiracetam: LEV) or GR agonist (dexamethasone: DEX) treatment on the subcellular localization of various protein targets crucial for drug metabolism/transport and BBB integrity, HBMECs, HBMEC+HA-GRβ, and EPI-ECs were divided into four treatment groups each: vehicle control, OXC (25 µg/mL), LEV (15 µg/mL), and DEX (10 µM) for 24 h. The cells were fractionated into cytoplasmic and nuclear fractions using the NE-PER Nuclear and Cytoplasmic Extraction Reagents kit as described above (Thermo Fisher Scientific, cat. 78833) at 6 h (HBMEC and HBMEC+ HA-GRβ) and 24 h (HBMEC, HBMEC+HA-GRβ, and EPI-EC) and analyzed by Western blot. Protein concentration was estimated by the Bradford method. Cell culture supernatant samples were also collected at each time point.

### 4.10. Determining MMP Activity by Zymography

MMP activity was determined by gelatin zymography using the samples obtained from the endothelial cell supernatant with and without drug treatment. A total of 20 µL of each sample was loaded into gelatin zymography gels (Thermo Fisher Scientific, cat. ZY00102BOX) and run at 100 V for about 2 h [45]. The gels were then incubated in renaturing buffer (2.5% Triton-X 100 in distilled water) for 30 min at room temperature followed by 1× developing buffer (Thermo Fisher Scientific, cat. LC2671) for 30 min at room temperature to equilibrate the gels. Then, the gels were incubated at 37 °C in 1× developing buffer for 18 h. Gels were stained with 0.5% Coomassie Brilliant Blue R-250 (Bio-Rad Laboratories, cat. 161-0400) prepared in destaining solution (60% distilled water, 30% methanol, 10% acetic acid) for 30 min, and cleared with destain solution for 30 min to 1 h to visualize the bands before imaging. Images were processed and quantified densitometrically using ImageJ software. Origin Pro 9.0 Software was then utilized to identify significant differences in MMP activity.

### 4.11. Data Analysis and Statistics

Origin Pro 9.0 Software was used for data analysis and statistical interpretation of data. Paired *t*-test was used to compare dysplastic and non-dysplastic brain tissue regions of the same individual patient. One-way or two-way analysis of variance (ANOVA) was utilized to compare multiple groups, with a Tukey post hoc test. All data are presented as mean with standard deviation (SD), and *p* < 0.05 was considered to be statistically significant.

## Figures and Tables

**Table 1:** Demographic details.

ID#	Age (Yrs)	Sex	ASMs	CYP- Mediated ASMs	Seizure Freq. (Per Week)	Duration of Epilepsy (Yrs)	Resected Region	Pathology Details	Exp. Use
62	28	F	CLB LEV OXC	CLB OXC	1 ± 1	22	Left mesial frontal lobe	FCD; Mild focal perivascular chronic inflammation; Focal subpial gliosis; Focal perivascular white matter atrophy	IHC, WB
44	13	M	LTG ZNS	ZNS	3 ± 1	9	Right frontal lobe	FCD; Nodular heterotopia; Perivascular chronic inflammation; Perivascular white matter atrophy, subpial gliosis	WB
46	34	F	LEV TPM	TPM	1	26	Right frontal lobe	FCD; Perivascular chronic inflammation; focal changes consistent with remote infarct/contusion damage; Subpial gliosis	WB
28	64	F	TPM LEV OXC	TPM OXC	1	27	Left frontal lobe	Focal cortical architectural disorganization consistent with FCD; Microcalcification; Focal perivascular white matter atrophy; Mild focal perivascular inflammation	IHC, WB
61	10	M	CLB LTG FBM	CLB	1	8	Right superior frontal lobe	FCD; Rare perivascular chronic lymphocytic inflammation; No balloon cells	IHC, WB
66	25	F	LTG BRV		7	3	Left frontal lobe	Cortex with minimal non-specific findings and no large dysmorphic neurons; Architecture minimally distorted	WB
51	1	F	VGB LCM	LCM	2 ± 1	1	Left frontal lobe	FCD; Mild focal perivascular chronic inflammation	WB
53	24	M	CBZ ZNS	CBZ ZNS	4 ± 1	19	Right occipital lobe	FCD; Focal perivascular white matter atrophy; Mild focal perivascular chronic inflammation; Subpial gliosis	WB
56	16	F	LCM PER RUF	LCM PER RUF	Not noted	15	Left occipital lobe/temporal lobe	FCD; Mild focal cortical dysplasia, marked neuronal loss and gliosis	WB
38	29	F	PHT ZNS CLB	PHT ZNS CLB	14 ± 2	24	Left temporal lobe/frontal lobe	FCD, Mild focal cortical architectural disorganization; Mild focal perivascular chronic inflammation	WB
41	13	F	OXC CLB CLZ	OXC CLB CLZ	1	14	Right frontal lobe	FCD, Focal architectural disorganization and Subpial gliosis	WB
43	32	M	OXC ZNS	OXC ZNS	5 ± 11	30	Left parietal lobe	FCD, Mild focal cortical architectural disorganization; Subpial gliosis; Contusional damage; Perivascular chronic inflammation	WB
76	4	M	LCM CLZ	LCM CLZ	7 ± 1	3	Left lateral temporal lobe	Changes consistent with remote infarcts/ischemic damage with microcalcification; Gliosis; Focal giant cells; Focal chronic inflammation	WB
26	27	M	LTG ZNS	ZNS	1	11	Left frontal lobe	Mild focal cortical architectural disorganization; Focal changes consistent with remote ischemic damage; Subpial gliosis; Meningeal fibrosis with perivascular and meningeal chronic inflammation	WB
42	20	F	LTG CLB	CLB	10 ± 2	9	Left lateral temporal lobe	FCD, Focal architectural disorganization and Subpial gliosis	WB
33	37	M	LTG LCM	LCM	1	15	Right frontal lobe	FCD, Focal cortical architectural disorganization; Subpial gliosis; Focal contusional damage	WB
29	47	F	ZNS CITA	ZNS CITA	Not noted	Not noted	Left lateral temporal lobe	Focal subpial gliosis	WB
80	55	F	OXC LTG	OXC	3	7	Temporal lobe	Mild cortical architectural abnormality; Mild diffuse subpial gliosis; Mild perivascular fibrosis	WB
86	62	M	PHT ZNS	PHT ZNS	1	62	Left temporal lobe	Diffuse subpial gliosis; Microscopic subacute infarct-like foci (gliosis and macrophages)	WB
88	49	M	OXC LEV ZNS CLZ LOR	OXC ZNS CLZ	1 every 2 months	2	Left frontal lobe	Mild focal cortical architectural disorganization consistent with FCD; Focal changes consistent with contusional damage/infarct; Meningeal chronic inflammation; Subpial gliosis	WB
142	23	M	LCM CLB	LCM CLB	0.75	2	Left anterior temporal lobe	FCD	WB
167	42	F	CLB LTG	CLB	1 per month	9	Right temporal lobe	Mild focal cortical architectural disorganization consistent with FCD; Mild focal perivascular white matter atrophy; Subpial gliosis	WB
160	20	F	LTG LCM MDZ	LCM MDZ	1 per month	18	Right temporal lobe	Mild focal cortical architectural disorganization consistent with FCD; Mild focal perivascular white matter atrophy; Subpial gliosis	WB
9	22	M	PHT CLB LTG	PHT CLB	1 per month	18	Right frontal lobe	Focal cavitary changes and gliosis consistent with focal infarct/contusional damage; Focal perivascular white matter atrophy; Subpial gliosis	Cell culture WB
111	35	M	ZNS CLB CLZ	ZNS CLB CLZ	3 ± 1	8	Right temporal lobe	Mild focal cortical architectural disorganization suggestive of FCD; Perivascular white matter atrophy; Mild perivascular chronic inflammation; Focal subpial gliosis	Cell culture WB

Abbreviations: Yrs: years; ASM: Antiseizure medications; F: Female; M: Male; LEV: levetiracetam; OXC: oxcarbazepine; TPM: topiramate; BRV: brivaracetam; ESL: eslicarbazepine acetate; CLB: clobazam; LTG: lamotrigine; ZNS: zonisamide; VGB: vigabatrin; LCM: lacosamide; CBZ: carbamazepine: PER: perampanel; RUF: rufinamide; LCR: levocarnitine; FBM: felbamate; CLZ: clonazepam; DZP: diazepam; GBP: gabapentin; LOR: lorazepam; MDZ: midazolam; PHT: phenytoin; FCD: focal cortical dysplasia; Exp. use: experimental use; IHC: immunohistochemistry; WB: Western blot.

## Data Availability

Data that do not compromise ethical standards and patient confidentiality will be available upon reasonable request.

## Supplementary Materials

The following supporting information can be downloaded at: https://www.mdpi.com/article/10.3390/ijms23094940/s1.
