# Supplementary material for: Glucocorticoid Receptor β Isoform Predominates in the Human Dysplastic Brain Region and Is Modulated by Age, Sex, and Antiseizure Medication
Source: Int J Mol Sci. 2022 Apr 29;23(9):4940. doi: 10.3390/ijms23094940 (PMC9099578; doi:10.3390/ijms23094940)
Supplement: Supplementary file 1 [file ijms-23-04940-s001.zip › ijms-1695389-supplementary.pdf]

**Supplementary Material**

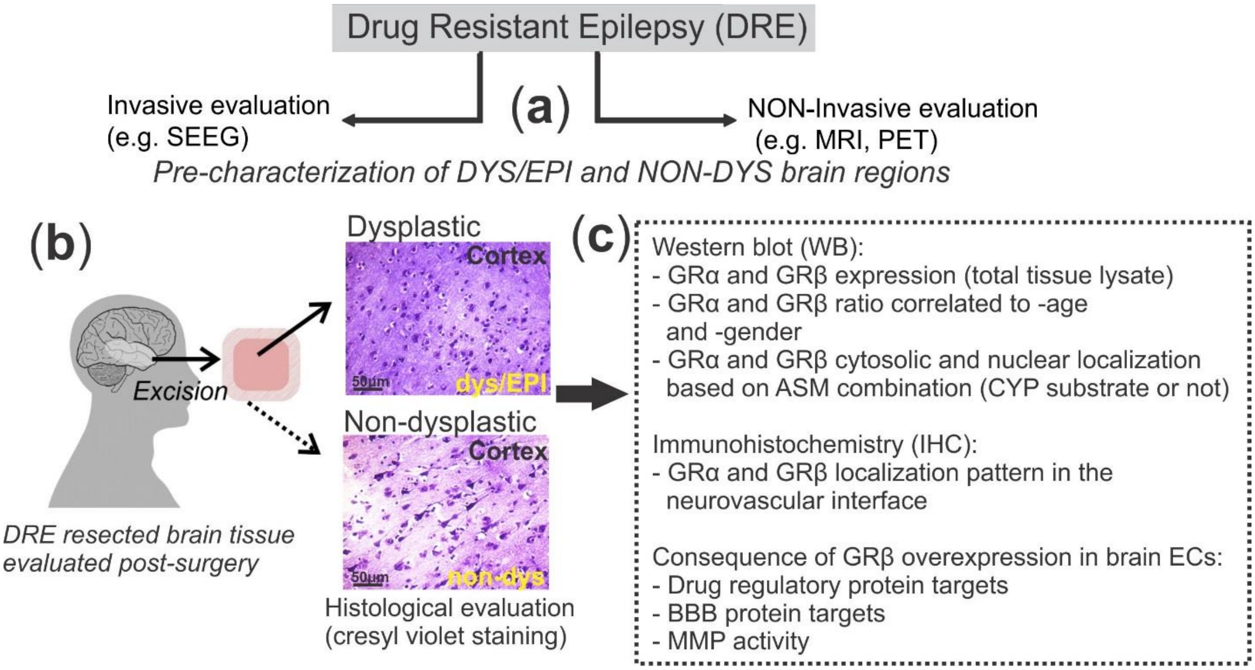


**Figure S1.** Experimental design. (**a**) The pre-operative characterization performed independently of the project by physicians allows for determination of dysplastic and non-dysplastic regions from the resected brain tissue. (**b**) These tissues were then characterized by histopathological staining post-resective surgery and were (**c**) used for evaluation of various endpoints for this study.

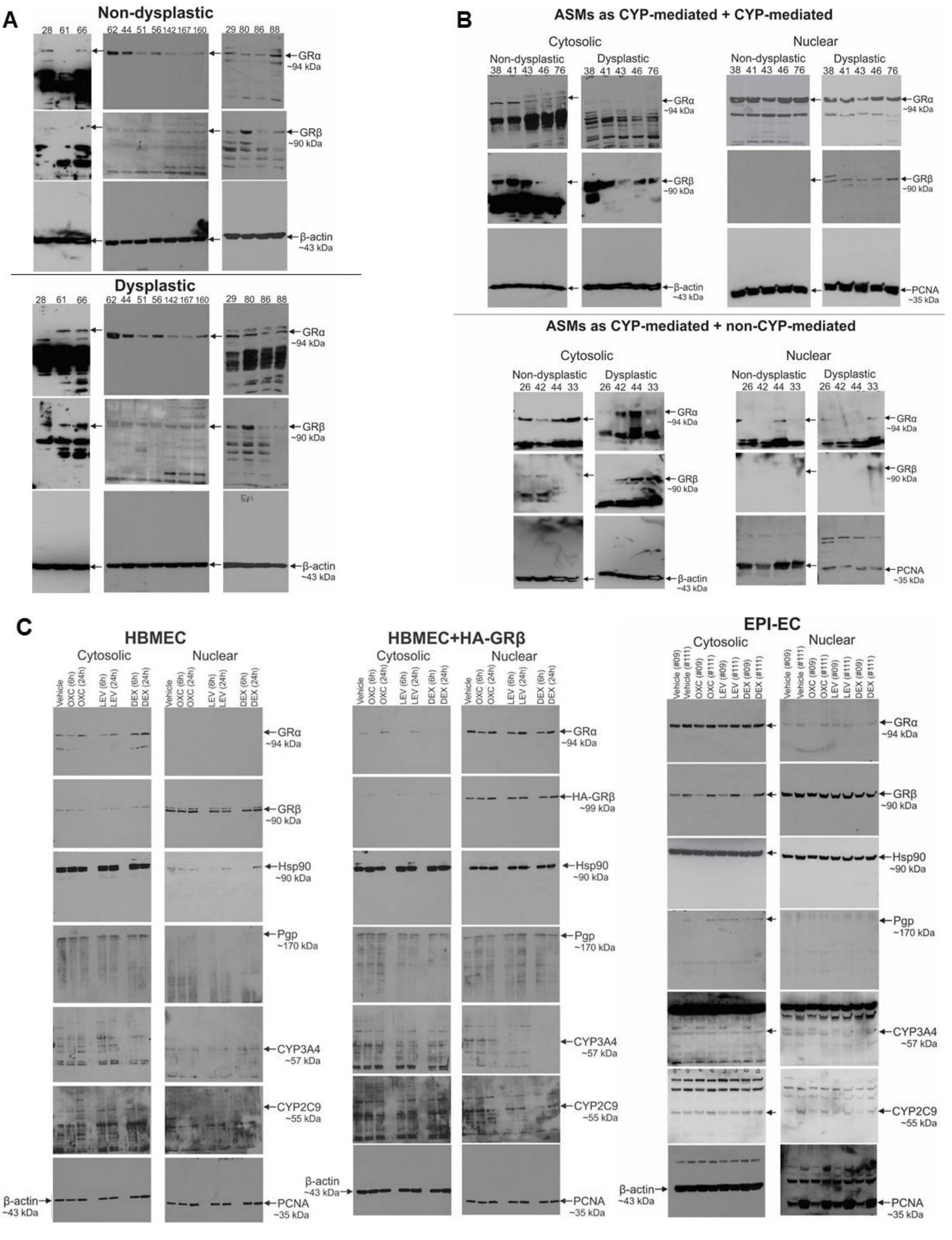

A
Non-dysplastic
28 61 66
62 44 51 56 142 167 160
29 80 86 88
GRα ~94 kDa
GRβ ~90 kDa
β-actin ~43 kDa
Dysplastic
B
ASMs as CYP-mediated + CYP-mediated
Cytosolic
Nuclear
38 41 43 46 76
PCNA ~35 kDa
ASMs as CYP-mediated + non-CYP-mediated
26 42 44 33
C
HBMEC
HBMEC+HA-GRβ
EPI-EC
Vehicle OXC (6h) OXC (24h) LEV (6h) LEV (24h) DEX (6h) DEX (24h)
Vehicle (#09) Vehicle (#111) OXC (#09) OXC (#111) LEV (#09) LEV (#111) DEX (#09) DEX (#111)
HA-GRβ ~99 kDa
Hsp90 ~90 kDa
Pgp ~170 kDa
CYP3A4 ~57 kDa
CYP2C9 ~55 kDa

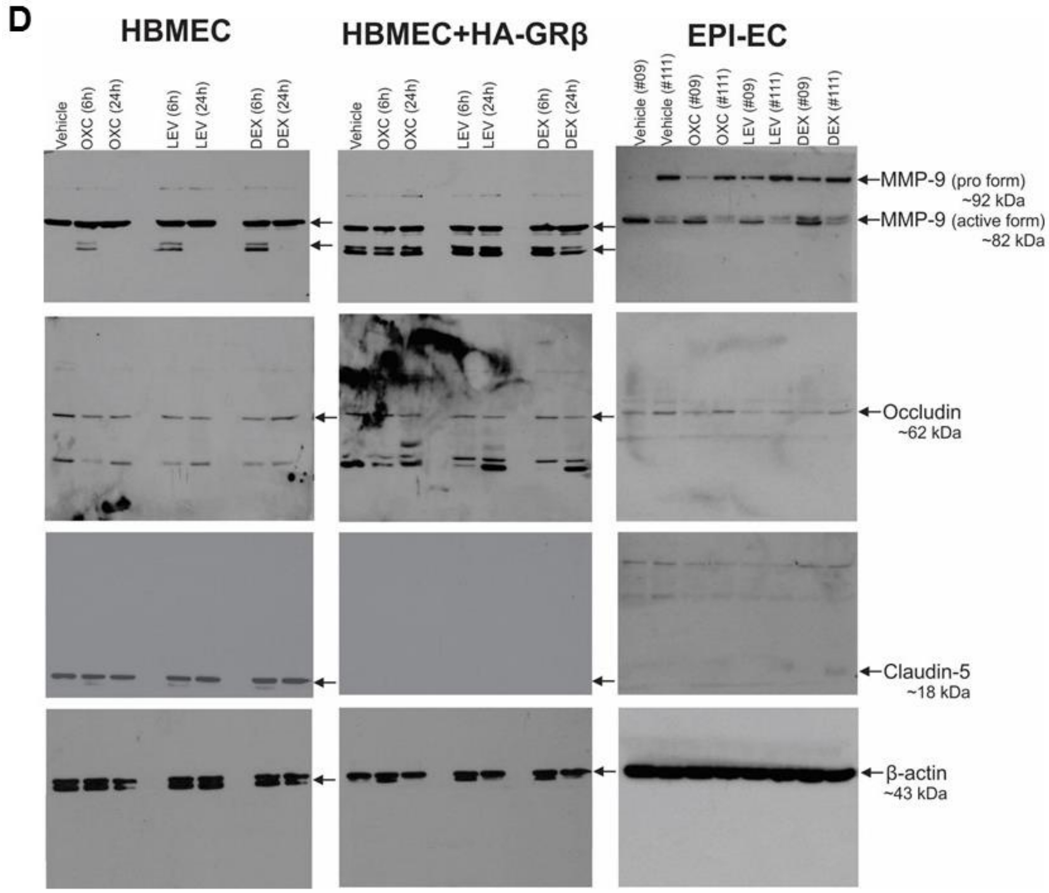


**Figure S2.** Full representative western blots.

**Table S1.** List of antibodies used for immunohistochemistry (IHC) and western blot (WB).

(**a**)

| *Primary Antibody* | *Host* | *Concentration* | *Manufacturer* | *Catalog No.* |
|---|---|---|---|---|
| *GRα* | Rabbit | WB: 4μg/10mL<br>IHC: 1:100 | Invitrogen, Thermo Fisher Scientific, Carlsbad, CA | PA1-516 |
| *GRβ* | Rabbit | WB: 1:250<br>IHC: 1:100 | Invitrogen, Thermo Fisher Scientific, Carlsbad, CA | PA3-514 |
| *Hsp90* | Mouse | WB: 1:1500 | Origene | TA500494 |
| *MDR1/Pgp* | Mouse | WB: 1:100 | Calbiochem | 517310 |
| *CYP3A4* | Rabbit | WB: 1:800 | Abcam | ab3572 |
| *CYP2C9* | Rabbit | WB: 1:100 | Abgent | AP7881c |
| *MMP-9* | Mouse | WB: 1:1000 | Sigma-Aldrich, St. Louis, MO | SAB5200294 |
| *Claudin-5* | Mouse | WB: 1:500 | Invitrogen, Thermo Fisher Scientific, Carlsbad, CA | 35-2500 |
| *Occludin* | Rabbit | WB: 1:250 | Invitrogen, Thermo Fisher Scientific, Carlsbad, CA | 71-1500 |
| *NeuN* | Mouse | IHC: 1:500 | MilliporeSigma, Burlington, MA | MAB377 |
| *GFAP* | Mouse | IHC: 1:100 | Sigma-Aldrich, St. Louis, MO | G3893 |
| *β-actin* | Mouse | WB: 1:10,000 | Sigma-Aldrich, St. Louis, MO | A1978 |
| *PCNA* | Rabbit | WB: 1:1000 | Proteintech Group | 10205-2-AP |

(**b**)

| *Secondary Antibody* | *Concentration* | *Manufacturer* | *Catalog No.* |
|---|---|---|---|
| *FITC Donkey α-Rabbit* | IHC: 1:100 | Jackson Immunoresearch, West Grove, PA | 711-C95-152 |
| *Alexa 594 Donkey α-Mouse* | IHC: 1:100 | Jackson Immunoresearch | 715-585-150 |
| *Biotinylated Goat α-Rabbit-IgG* | IHC: 1:200 | Vector Laboratories, Inc. Burlingame, CA | BA-1000 |
| *Polyclonal Goat Anti-Rabbit IgG HRP* | WB: 1:2500 | Dako, part of ThermoFisher Scientific Waltham, MA | P0448 |
| *Polyclonal Goat Anti-Mouse IgG HRP* | WB: 1:2500 | Dako, part of ThermoFisher Scientific Waltham, MA | P0447 |
